# A Novel RP-HPLC-DAD Method Development for Anti-Malarial and COVID-19 Hydroxy Chloroquine Sulfate Tablets and Profiling of In-Vitro Dissolution in Multimedia.

**DOI:** 10.21203/rs.3.pex-880/v2

**Published:** 2020-05-05

**Authors:** THIRUPATHI DONGALA, Santhosh Kumar Ettaboina, Naresh Kumar Katari

**Affiliations:** Aurex Laboratories LLC; GITAM deemed to be University

**Keywords:** Anti malaria, COVID-19, Hydroxy chloroquine sulfate, Dissolution, validation

## Abstract

Hydroxychloroquine sulfate is one of a large series of 4-aminoquinolines with antimalarial activity. Moreover, it is used for the treatment of rheumatoid arthritis. Sometimes Hydroxychloroquine sulfate is very effective for the treatment of autoimmune diseases. Based on the recent clinical experiments it is exploiting for the treatment of COVID-19, corona virus across the globe. A Reverse phase RP-HPLC method have been developed and validated as per the current ICH guidelines for estimation of Hydroxychloroquine sulfate tablets. As part of method validation specificity, linearity, precision and recovery parameters were verified. The concentration and area relationships were linear (R^2^ > 0.999), over the concentration range of 25 to 300 μg mL^−1^ for HCQ. The relative standard deviations for precision and intermediate precision were less than 1.5%. The proposed RP-HPLC generic method was applied successfully for evaluation of invitro dissolution profile with different pH conditions like 0.1N HCl, pH 4.5 Acetate buffer and pH 6.8 Phosphate buffers of US marketed reference product.

## Introduction

Hydroxychloroquine (HCQ) is solid oral prescription tablets formulations that have been used for medication of malaria and a wide variety of inflammatory conditions. Moreover, HCQ has in-vitro activity for the treatment of SARS CoV-2, and related coronaviruses problems [1–3]. In recent days it is proven to have superficial ability and tolerable safety against COVID-19 (coronavirus-2019) related pneumonia in clinical experiments performed in China [4]. The COVID-19 (corona virus) 2019, appeared in middle of December 2019, has proliferated swiftly, now confirmed in numerous countries across the globe. As of March 01, 2020, the COVID-19 has produced 860,076 infections and 42297 deaths in all countries majorly USA, Italy, Spain, China and other countries across the globe etc.,

After conducting the multiple clinical trails by the State Council of China, confirmed that chloroquine phosphate, a long-standing medicine used for treatment of malaria, had proved noticeable efficacy and satisfactory safety in treating COVID-19 (Corona virus 2019) related pneumonia patients. Serious unfavorable reactions to HCQ and chloroquine phosphate were not observed in the COVID-19 patients. Based on these conclusions, a conference was organized on 15, February 2020; members including professionals and experts from government and regulatory authorities of clinical trials agreed and approved that HCQ and chloroquine phosphate has effective against COVID-19. Based upon limited available clinical trials data, HCQ is recommending for treatment of COVID-19 infected patients in most of the countries. Due to wider accessibility of these HCQ tablets in the United States, it has been administered to hospitalized COVID-19 patients. It is still under investigation clinical trials for treatment of patients with different stages like mild, moderate, and severe COVID-19. The chemical structure of HCQ was shown in Fig. 1.

Currently, most of the countries were looking for HCQ drug product literature and other related information for better understanding, due to the COVID 19 pandemic. It is very important to know the drug product release with respective the time in invitro conditions. The literature survey revealed different types of analytical techniques are used for determination of HCQ to report the information, such as HPLC with PDA detector [5–6], HPLC with UV detector [7–11], chromatography-tandem mass spectrometry [12–13], LC/IT/MS [14], capillary-LC [15], Electrochemical study [16].

Majority of the literature was reported in pharmacokinetics of HCQ in biological fluids. Only one method in the USP monograph was reported with UV-Spectro photometric method for estimation of HCQ tablets in vitro dissolution profile. Generally, UV methods results are not reliable due to reproducibility problem. Other than USP method there was no literature on the HCQ tablets invitro dissolution profile in different multimedia to understand the product behavior. The RP-HPLC is powerful and simple technique for quantification drug products in different pH conditions. Moreover, in the regulated pharmaceutical industry, the drug products released into the global market after determination of quality safety and efficacy. Hence, HPLC methods are required with suitable precision, accuracy and sensitivity level. The main aim of present work is to develop a simple and reproducible HPLC method for estimation of HCQ dissolution profile (0.1N HCl, 0.01N HCl, pH 4.5 sodium acetate, pH 6.8 phosphate buffer).

## Reagents

Procured the HCQ with certified purity of 99.2% from the SCI pharma. AR grade ortho phosphoric acid, Hydrochloric acid, potassium dihydrogen phosphate and acetic acid procured were from VWR chemicals, Radnor, PA, USA. HPLC grade acetonitrile (99.9%) and methanol from J.T. Baker procured from VWR chemicals, Radnor, PA, USA. High quality HPLC grade water used.

## Equipment

The Agilent HPLC 1260 Infinity-II consists four channels, pressure range up to 600 bar, degasser with integrated purge valve, thermostatic sampler and column compartment. The photo diode array detector (PDA) connected to empower 3 software (Build 3471 SPs Installed: Feature Release 3 DB ID: 2639633283) to monitor the output signal. The dissolution apparatus was Disteck Premiere 5100 model. The column is Agilent Zorbax C8, 250 × 4.6 mm i.d., 5μm. Sartorius semi micro and micro balances used for weighing of impurities, standards and samples. Bio-Technics ultra sonicator used to extract drug from the sample matrix.

## Procedure

### Chromatographic conditions

The chromatogram was achieved with in 5 min run time by using Agilent, Zorbax C8, 250 × 4.6 mm i.d., column with isocratic method. The Mobile Phase consists 0.01M of 1-pentane sulfonic acid and 0.02% of Ortho phosphoric acid in purified water with acetonitrile and methanol (800: 100: 100 v/v).

The flow rate was 1.0 mL min^−1^ and injection volume were 10 μl. Detection was made at 254 nm by using dual absorbance detector (DAD).

### Preparation of dissolution media Buffer

To prepare 0.1N Hydrochloric acid, transferred 8.5ml of Conc. HCl (35%) in to 1000ml of volumetric flask and made up to volume with water. For pH 4.5 Sodium acetate buffer, weighed and transferred 2.99g of sodium acetate (NaC_2_H_3_O_2_.3H_2_O) in a 1000ml volumetric flask added 14ml of 2N acetic acid (CH_3_COOH) and made up to volume with water. To prepare pH 6.8 phosphate buffer, transferred 250mL of 0.2 M monobasic potassium phosphate solution and 112 mL of 0.2 M NaOH in to 1000ml volumetric flask and made up to volume with water.

### Dissolution conditions and preparation of sample:

To evaluate the dissolution of profile of HCQ reference listed product (RLD) (Plaquenil) with respective the time used USP apparatus-II (Paddle) having 50 rpm and 900mL of medium maintained 37.0°C temp. After dropping the RLD tablets in to six dissolution vessels collected the samples at different time points. and injected in to HPLC.

## Anticipated Results

For the estimation of HCQ tablets started method development with selection of buffer, we selected %1-pentane sulfonic acid and 0.02%of OPA in purified water. Mixed the buffer with methanol and acetonitrile in the ratio of 800:100:100 v/v for the better peak shape. Selected, Agilent Zorbax C8, 250 × 4.6 mm i.d column for estimation of HCQ for better system suitability parameters like tailing factor and theoretical plates. The flow rate of HPLC was set 1.0 mL min^−1^ and injection volume was 10 μl. HPLC Detection was made at 254 nm by using dual absorbance detector (DAD). Evaluated system suitability parameters (retention time, tailing factor and theoretical plates) at 254 nm by using DAD detector (Fig. 2). As part of method validation specificity, linearity, precision and recovery parameters were verified. The concentration and area relationships were linear (R^2^ > 0.999), over the concentration range of 25 to 300 μg mL^−1^ for HCQ. The relative standard deviations for precision and intermediate precision were less than 1.5%. The accuracy for 50% to 150% dissolution were found to more than 97%. The proposed RP-HPLC generic method was applied for analysis of US marketed products successfully.

Performed invitro dissolution profile for the Reference listed drug and in-house formations to compare the product release with respective the specified time intervals. During the dissolution profile we were selected four different media like 0.1N HCl, pH 4.5 acetate buffer, pH 6.8 phosphate buffer to understand the product behaviors in low to high pH conditions. The results were shown different drug release pattern up to first 15–20 minutes of time. After that all the media were reached optimum release (Table -1 & Figure 3).

### Conclusion:

The hydroxychloroquine is currently recommending for treatment of hospitalized COVID-19 patients in most of the countries in the world. It is very important to understand the drug profiling invitro conditions, Hence a simple HPLC method has been developed successfully for estimation of HCQ dissolution profile in invitro conditions. The optimized HPLC method used for estimation of dissolution profile of RLD and in-house tablets. The optimized method was validated as per the ICH guidelines and the results were found satisfactory. Finally developed method was used in the quality control lab for the analysis of dissolution.

## Supplementary Material

Supplement Table 1: Dissolution profile of HCQ RLD and In house formulations results

Supplement Figure 1: Chemical strucre of Hydroxy chloroquine sulfate
Figure 2: Typical Chromatogram of Hydroxy chloroquine sulfate standard
Figure 3: Graph model comparison of HCQ dissolution profile of Reference Vs In House
