# Supplementary material for: A Novel RP-HPLC-DAD Method Development for Anti-Malarial and COVID-19 Hydroxy Chloroquine Sulfate Tablets and Profiling of In-Vitro Dissolution in Multimedia
Source: Res Sq. 2020 May 5:rs.3.pex-880. Preprint. [Version 2] doi: 10.21203/rs.3.pex-880/v2 (PMC7336707; doi:10.21203/rs.3.pex-880/v2)
Supplement: Supplement — Table 1: Dissolution profile of HCQ RLD and In house formulations results [file Tables.docx]

Table 1 : Dissolution profile of HCQ RLD and In house formulations results

| **Time** | **pH 4.5 Acetate Buffer** | | | | **pH 6.8 Phosphate Buffer** | | | | **0.1 N HCl** | | | |
| --- | --- | --- | --- | --- | --- | --- | --- | --- | --- | --- | --- | --- |
|  | **Reference** | | **In-House** | | **Reference** | | **In-House** | | **Reference** | | **In-House** | |
|  | **%** | **% RSD** | **%** | **% RSD** | **%** | **% RSD** | **%** | **% RSD** | **%** | **% RSD** | **%** | **% RSD** |
| **5-min** | 25 | 36 | 45 | 18.4 | 19 | 37.6 | 30 | 16.4 | 23 | 23.9 | 29 | 14.1 |
| **10-min** | 60 | 21.3 | 78 | 8.8 | 50 | 17.3 | 55 | 16.6 | 51 | 17.2 | 57 | 16.5 |
| **15-min** | 80 | 13.8 | 88 | 6.8 | 75 | 12.1 | 71 | 15.8 | 73 | 14 | 75 | 13.1 |
| **20-min** | 90 | 7.7 | 93 | 4 | 88 | 5.9 | 80 | 13.8 | 85 | 10 | 86 | 8.7 |
| **30-min** | 96 | 3.2 | 94 | 3 | 94 | 2.6 | 86 | 11.5 | 92 | 3.6 | 93 | 3.9 |
| **45-min** | 97 | 2.1 | 96 | 2.4 | 95 | 1.6 | 87 | 10.1 | 94 | 1.2 | 97 | 1.8 |
| **60-min** | 97 | 1.6 | 97 | 2 | 95 | 1.6 | 89 | 9.1 | 94 | 1.2 | 98 | 1.2 |
| **Recovery** | 98 | 1.2 | 98 | 1.7 | 96 | 1.9 | 98 | 1.2 | 95 | 0.9 | 98 | 1.2 |
