# Supplementary material for: A Novel RP-HPLC-DAD Method Development for Anti-Malarial and COVID-19 Hydroxy Chloroquine Sulfate Tablets and Profiling of In-Vitro Dissolution in Multimedia
Source: Res Sq. 2020 May 5:rs.3.pex-880. Preprint. [Version 2] doi: 10.21203/rs.3.pex-880/v2 (PMC7336707; doi:10.21203/rs.3.pex-880/v2)
Supplement: Supplement — Figure 1: Chemical strucre of Hydroxy chloroquine sulfate Figure 2: Typical Chromatogram of Hydroxy chloroquine sulfate standard Figure 3: Graph model comparison of HCQ dissolution profile of Reference Vs In House [file Figures.docx]

Figure 1: Chemical strucre of Hydroxy chloroquine sulfate

Figure 2: Typical Chromatogram of Hydroxy chloroquine sulfate standard


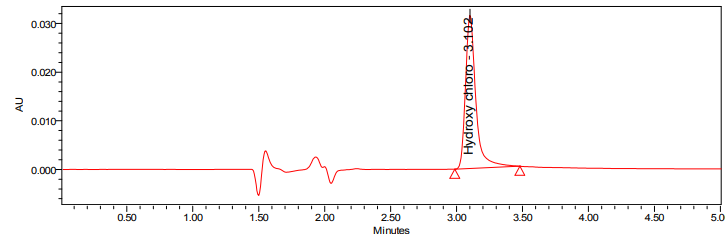


Figure 3: Graph model comparison of HCQ dissolution profile of Reference Vs In House
